# Comparing optical and electromagnetic tracking systems to facilitate compatibility in sports kinematics data

**DOI:** 10.1080/23335432.2021.2003719

**Published:** 2021-11-21

**Authors:** Caryn A. Urbanczyk, Alessandro Bonfiglio, Alison H. McGregor, Anthony M.J. Bull

**Affiliations:** a Department of Bioengineering, Imperial College London, London, UK; b Department of Surgery & Cancer, Imperial College London, London, UK

**Keywords:** Optical motion capture, electromagnetic motion tracking, kinematics, rowing

## Abstract

Electromagnetic (EM) tracking has been used to quantify biomechanical parameters of the lower limb and lumbar spine during ergometer rowing to improve performance and reduce injury. Optical motion capture (OMC) is potentially better suited to measure comprehensive whole-body dynamics in rowing. This study compared accuracy and precision of EM and OMC displacements by simultaneously recording kinematics during rowing trials at low, middle, and high rates on an instrumented ergometer (n=12). Trajectories calculated from OMC and EM sensors attached to the pelvis, lumbar spine, and right leg were highly correlated, but EM tracking lagged behind ergometer and OMC tracking by approximately 6%, yielding large RMS errors. When this phase-lag was corrected by least squares minimization, agreement between systems improved. Both systems demonstrated an ability to adequately track large dynamic compound movements in the sagittal plane but struggled at times to precisely track small displacements and narrow angular ranges in medial/lateral and superior/inferior directions. An OMC based tracking methodology can obtain equivalence with a previously validated EM system, for spine and lower limb metrics. Improvements in speed and consistency of data acquisition with OMC are beneficial for dynamic motion studies. Compatibility ensures continuity by maintaining the ability to compare to prior work.

## Introduction

Optical and electromagnetic motion capture systems are frequently employed to measure whole body kinematics in biomechanics research. These systems each have their own advantages and drawbacks. Optical motion capture (OMC) uses infrared cameras to record the position of reflective markers placed on the body, whereas electromagnetic tracking systems (EM) utilize receivers within an electromagnetic field to compute position and orientation of body segments in space. EM tracking provides numerous advantages in sports and clinical biomechanics, including relatively simple digitization, ability to record and display position and orientation with little data processing, and 6 degree of freedom sensing, without direct line-of-sight requirement between transmitter and receivers (Parent 2012; Franz et al. 2014).

Collaboration between Imperial College and British Rowing has used the ‘Flock of Birds’ (Ascension Technologies, USA) EM tracking system lower extremity and lumbar spine biomechanics research on elite rowers, offering a framework to describe proper sequencing (Bull et al. 1998; Bull and McGregor 2000; McGregor et al. 2004) and establishing links between biomechanics parameters and performance metrics, which continue to be used to analyze quality of elite rowing technique (Buckeridge et al. 2016, 2015; Murphy 2009). However, current generation EM systems still present drawbacks, which relate to the functional range of accuracy of the magnetic field, a frame rate affected by the number of receivers (LaScalza et al. 2003), any restricted movement from tethered cable connections causing a hindrance to subjects (Sorriento et al. 2020), a sensitivity to metal and other electronics (Meyer et al. 2008; Ng et al. 2009), and a smaller number of body segments that can be tracked due to limited number of physical sensors (Murphy et al. 2011). Most EM systems use a digitization and indirect tracking method wherein positions of digitized anatomical landmarks are defined relative to the EM sensors’ local coordinate system and used for building anatomical frames of body segments (Murphy 2009).

Optical motion technology presents advantages for whole-body tracking of dynamic activities in large capture volumes and has been successfully used to analyze posture and overall body movement during ergometer rowing (Pollock et al. 2009; Attenborough et al. 2012; Skublewska-Paszkowska et al. 2016). Despite a line-of-sight requirement between markers and cameras, OMC overcomes EM system limitations with increased frame rate (up to 240 Hz), direct tracking of anatomical landmarks using reflective markers and offers greater flexibility in marker placement (both individual and clusters). However, because orientation cannot be directly measured, more markers are required than with EM systems, and an OMC approach typically requires longer post-processing time for complex data (Parent 2012; Sorriento et al. 2020).

Advances in research methods and technologies reduce bias, improve precision, and expand measurement options, but at the cost of limiting comparability to data derived using alternative systems (Pueo and Jimenez-Olmedo 2017). While OMC systems have become the ‘gold standard’ method in motion capture (Corazza et al. 2010), there are studies using EM systems from a wide range of cohorts, and settings, whose records can be leveraged by developing a standard of interoperability between systems, including Imperial College’s wealth of historical biomechanics data on elite level rowers. This type of compatibility can be successfully used to preserve older data to operate with newer or different systems and facilitates interpretation of current and future OMC data by contextualization in relation to past EM measures (Rowlands et al. 2016).

Hassan et al. (2007) reported direct comparisons between OMC and EM tracking systems when receivers were affixed to a robotic articulating arm, providing an assessment of each system for slow movements with small angular deviations. They found that mean difference between each system and the robotic arm position did not exceed 2° but stressed the need for smoothing and rigid body correction in data processing. That study did not address the inaccuracies native to human subject testing, such as skin motion artifact, for which such rigid body corrections may be inappropriate. Few *in-vivo* studies have compared the performance of these systems for compound motions under dynamic conditions. Lugade et al. (2015) examined intra- and inter-day repeatability of EM and OMC measurements during a sit-to-stand task, finding high coefficients of multiple correlation (CMC) for sagittal plane hip, knee, and ankle joint angles and no differences between systems in joint range of motion (Lugade et al. 2015). There are no publications that assess these technologies in more complex, higher speed, whole-body motions, such as rowing.

The aims of this study were to analyze the accuracy and precision of EM and OMC displacements in three dimensions during ergometer rowing, quantifying the relative error in reported position of directly tracked receivers, digitized anatomical landmarks, and calculated joint centers.

## Methods

Twelve healthy subjects with at least 2 years of rowing experience participated in the study (11 female/1 male; age: 25.6 ± 2.2 years; height: 178.6 ± 7.8 cm; mass: 74.8 ± 6.0 kg). All subjects were club or national team athletes rowing regularly at the time of the study. Imperial College London research ethics committee granted approval, written informed consent was obtained from each participant prior to testing, and all athlete data was anonymized.

### Apparatus

**Figure 1.**
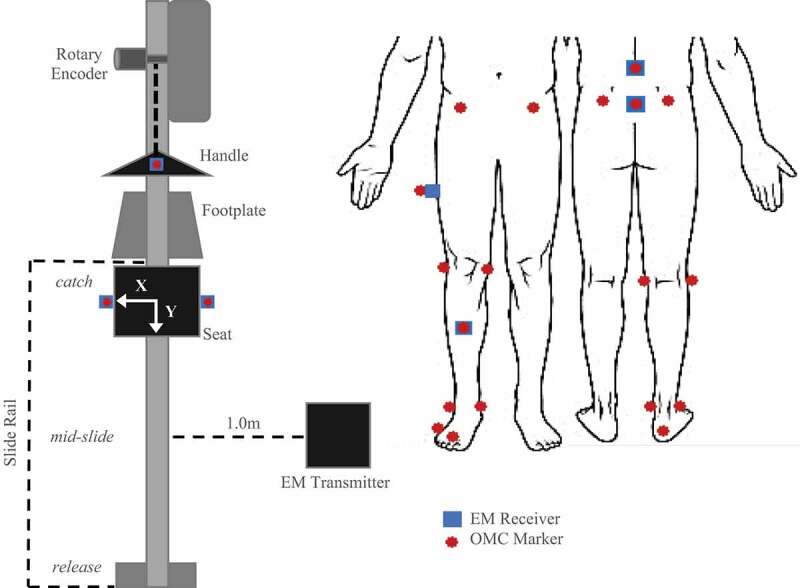
Diagram of OMC marker and EM receiver layout during rowing trials. Top-down view of sensors affixed to the instrumented ergometer (left). Anterior and posterior views of sensors as applied to subject anatomical landmarks (right). Relative position of key stroke occurrences (*catch, mid-slide, release*) is indicated to the left of the slide rail

External kinetics were recorded on a bespoke instrumented ergometer with load cells at the handle, seat, and footplates and a rotary encoder on the flywheel (Murphy 2009; Buckeridge et al. 2012). Kinematic data was simultaneously recorded with a ten-camera optical motion tracking system, operating at 100 Hz (‘MX T-series’, Vicon, Oxford, Uk), and a four receiver ‘extended range’ EM tracking system operating at 75 Hz (‘Flock of Birds’, Ascension Technologies, VT, USA). The lab was arranged for a large capture volume (36 m^3^). The EM system transmitter was located 1 m to the right of the ergometer slide rail and 1.25 m above the floor on a wooden tower (Figure 1). OMC data was streamed to Vicon Nexus 1.8.5 software, while ergometer outputs and EM data were streamed to a custom data acquisition program (LabView 2016, National Instruments, TX, USA). Real-time feedback was displayed to athletes and researchers during testing (McGregor et al. 2016).

### Protocol

#### Athlete tracking

OMC markers and EM receivers were attached to the pelvis, lumbar spine, and right leg of each subject (Figure 1). Four EM receivers were attached to the skin at the thoracolumbar (T12/L1) and lumbosacral junction (L5/S1) using adhesive pads and midway along the thigh and shank using fabric straps. EM system calibration followed the procedure previously described (Murphy et al. 2011), which involves digitizing relevant bony landmarks by palpation with an EM receiver attached to a stylus. Nine OMC markers were placed on the digitized bony landmarks as well as atop all four EM receivers and the ergometer handle (Figure 1). Digitization and marker placement was performed by a single operator, an experienced sports biomechanist with training in landmark palpation, skin marker placement and extensive experience with methods used in the current protocol.

Each rower performed three rowing trials at low, medium, and high stroke rates (18, 24, 28 strokes per minute, spm) on an instrumented ergometer (Urbanczyk et al. 2019, 2020). Real-time feedback from the ergometer monitor to the athlete facilitated maintaining a fixed stroke rate. Participants could rest as needed between each trial. Knee and ankle joint centers were defined as the midpoint between lateral and medial epicondyles and malleoli, respectively. Right hip joint center was determined from functional calibration (Camomilla et al. 2006).

#### Apparatus tracking

Three subjects participated in a secondary protocol, which involved tracking the ergometer apparatus under static and dynamic conditions but did not include marker placement on the athletes themselves. OMC and EM systems simultaneously recorded displacement of the ergometer handle and the ergometer seat – (left and right sides; Figure 1). OMC and EM measurements were compared to ergometer positions measured by the rotary encoder and a precision measuring tape during dynamic and static tracking, respectively.

### Data analysis and statistics

Synchronized motion data and external force data were processed in MATLAB (2017, MathWorks, MA, USA). Continuous rowing trials were smoothed using a 4^th^ order low-pass Butterworth filter with a 6-Hz cutoff frequency (Pollock et al. 2009) and divided into individual strokes where the *start* was identified as the minimum anterior/posterior (A/P) handle position. The *catch* was defined as the onset of handle force exceeding 75 N, with a steep, increasing slope. The *release* was defined as maximum A/P handle displacement. Each stroke was time normalized from 0–100% of completion using a cubic spline interpolation, such that the *drive* time was from *start* (0%) to *release*, and the *recovery* time was from *release* to a subsequent next *start* (100%) (Urbanczyk et al. 2019, 2020). All measurements shared a global coordinate frame, and each trial was referenced to initial marker/receiver position, permitting comparison of system drift between and within subjects. Statistics were run in RStudio (RStudio Team 2016) with a significance level of *α = 0.05*. Initial ANOVA analysis compared reported sensor position between stroke rates for each system; however, no significant difference was found as stroke rate increased. Therefore, data was pooled to reduce statistical complexity, with mean tracked position and 95% confidence intervals calculated across all speeds. Bland-Altman analysis was used to compare the bias and limits of agreement of each motion tracking system. Root mean square error (RMSE) was used to quantify the difference in each system’s tracked position to ground truth position during static testing. Geers metric (GM) was used to discriminate phase and magnitude differences in tracked position between systems during dynamic testing (Schwer 2007), where metric values of zero indicate perfect agreement, less than 0.30 difference is considered good and greater than 0.50 difference is considered poor (Schwer 2007; Klemt et al. 2019). Analysis of EM system lag and its correction used a method of least squares fitting, which retrieved the wavefront phase shift of the EM receivers relative to the OMC and ergometer systems. For every participant, and for each continuous 3-minute rowing trial, a single time-shift translational offset was calculated and applied to the EM system kinematic data as determined by the residuals of the minimization.

## Results

### Apparatus tracking: data consistency and capture latency

**Figure 2.**
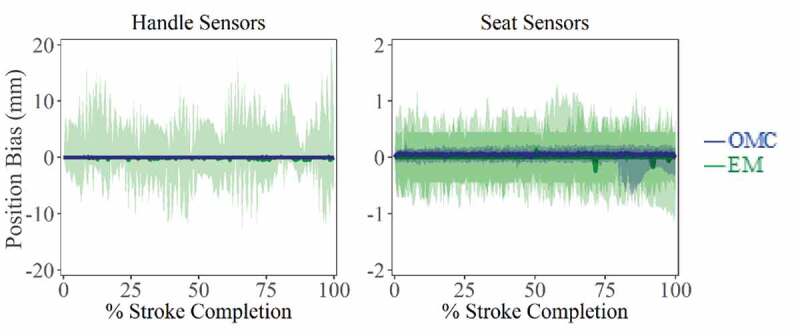
Difference from ground truth position of static marker/receivers in A/P plane for OMC and EM systems. Similar patterns were found for M/L and S/I directions

**Figure 3.**
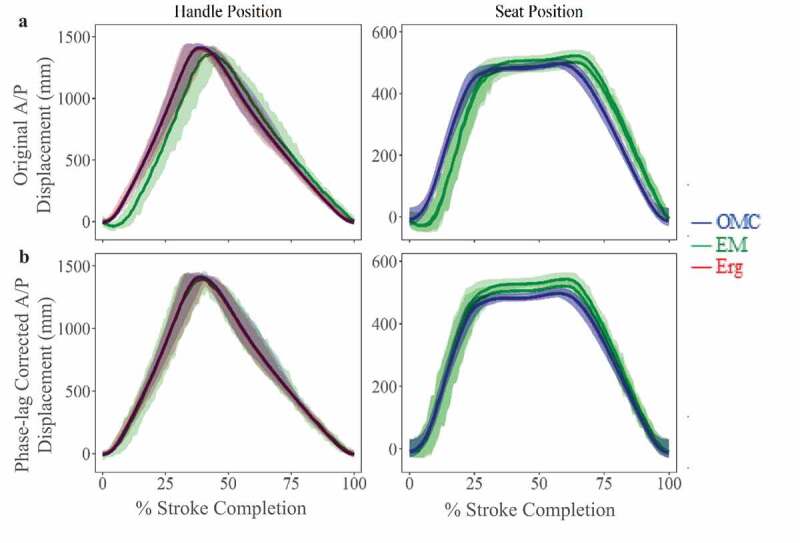
A/P plane displacements (mean ± 95% CI) for OMC and EM systems across all stroke rates. (a) Before phase-lag correction, EM system capture latency is visible as a rightward shift in mean displacement for handle and seat trajectories. (b) After phase-lag correction using least-squares fitting, differences between systems decreased, as indicated by smaller RMSE values

Static tracking of the handle and seat show that differences for both tracking systems, with respect to ground truth position, were similar (Figure 2). Average RMS error for the OMC system was 0.08 ± 0.04 mm, while EM system error was 1.44 ± 2.17 mm. Comparing variance of static measurements, the OMC system was much less prone to noise (*p < 0.001*), with 95% confidence intervals for the OMC system lying well within the limits of the EM system (Figures 2, 3). Under dynamic motion tracking, comparison of stroke trajectories captured by both tracking systems and the ergometer indicates that the lower frame rate and burst transmission of the EM system contribute to jagged tracking of smooth movements (Figure 3).

**Figure 4.**
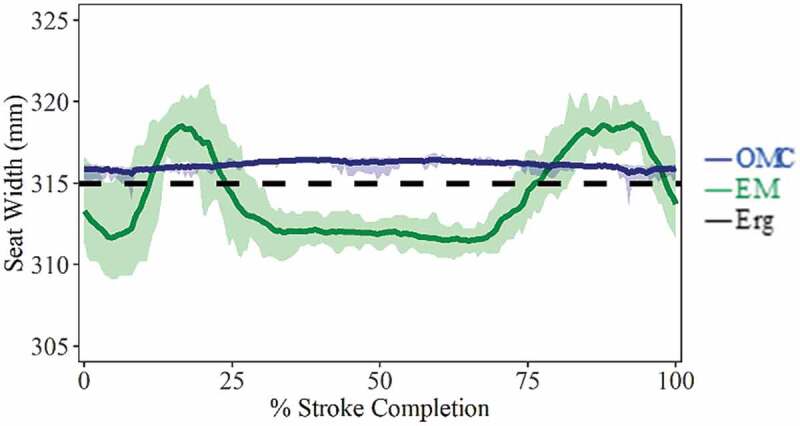
Seat width (mean ± SD), calculated as the M/L distance between markers/receivers fixed to left and right sides of ergometer seat. The OMC system slightly overestimates seat width throughout the stroke while the EM system was less consistent, underestimating seat width near the catch and release positions and overestimating seat width during mid-slide. Catch occurs at 0% and 100%. Release occurs at ~40%

Seat width, calculated from the distance between tracking markers/receivers on left and right sides of the ergometer seat, could be directly compared to the true measured seat width (315.0 mm), providing an estimate of system accuracy in the medial/lateral (M/L) direction (Figure 4). The OMC system slightly overestimates seat width in both static (315.6 ± 0.5 mm; RMS = 0.62 mm) and dynamic trials (316.1 ± 0.3 mm; RMS = 1.16 mm), while the EM system was less consistent during dynamic trials (314.1 ± 2.8 mm; RMS = 2.69 mm), underestimating seat width near the *catch* and *release* positions, where the receivers were radially farther from the transmitter and overestimating seat width when the receivers were closer to the transmitter during *mid-slide* (Figure 4). In static tracking, receiver position reported by the EM system has large errors and between-trial variance (290.3 ± 23.4 mm). At slide positions farther from the transmitter, seat width was underestimated (*catch*: 285.2 ± 0.3 mm, *release*: 260.4 ± 6.9 mm), but for *mid-slide* position closer to the transmitter, estimates were more accurate (*mid-slide*: 314.1 ± 1.8 mm).

Differences in A/P handle motion during dynamic apparatus tracking from each system were compared to handle position output measured using the rotary encoder directly on the ergometer. The EM showed significant differences in A/P handle position from both other systems (RMS = 136.2 mm; *p < 0.05*). The EM system lags both ergometer and OMC reported position by ~6% per stroke (Figure 3). There was no significant difference in measured A/P handle position between OMC and ergometer systems (RMS = 34.2 mm; *p = 0.93*). This apparent capture latency in the EM system tracking impacts time-related kinetic and kinematic relationships and any correlations or conclusions drawn therefrom. This capture latency could be corrected for by least squares minimization, compared to the known ergometer output positions. After latency correction, quality of fit between the EM system, the OMC system, and the ergometer improved substantially, with RMS error reduced from 136.4 mm to 34.4 mm (Figure 3).

### Athlete tracking: co-localized markers and joint centre estimates

**Figure 5.**
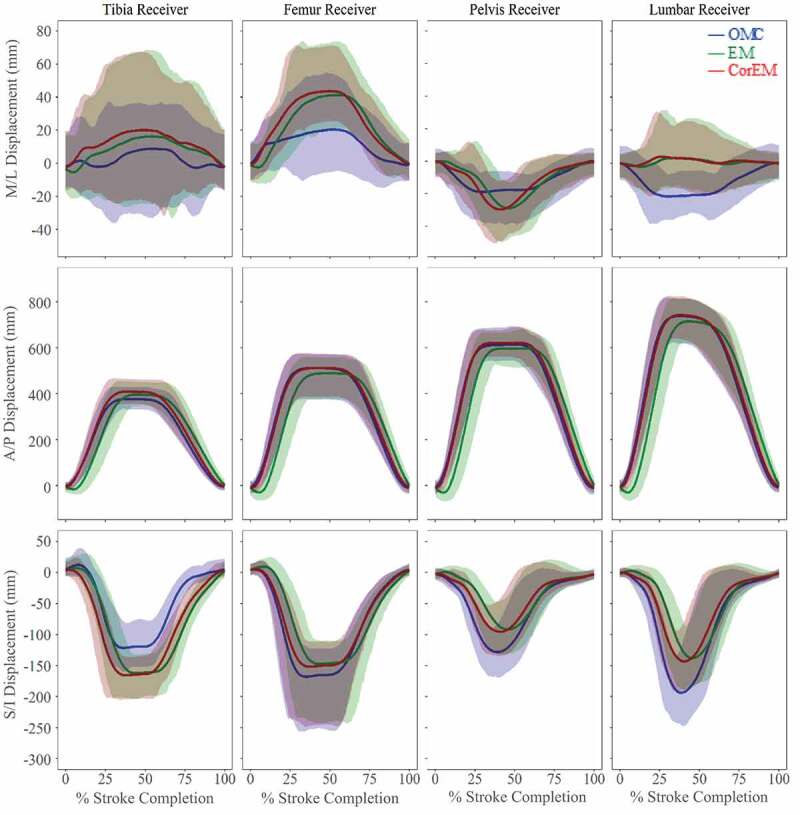
Co-localized marker tracking (mean ± 95% CI) in three dimensions with and without phase correction. These show similar trends despite EM capture latency, with each mean trajectory falling within well overlapped 95% confidence intervals. Agreement between systems in A/P displacements shows the most improvement with phase-lag correction. M/L and S/I displacements show more apparent magnitude differences between systems, but without a clear trend in over/under estimation of one system relative the other

**Figure 6.**
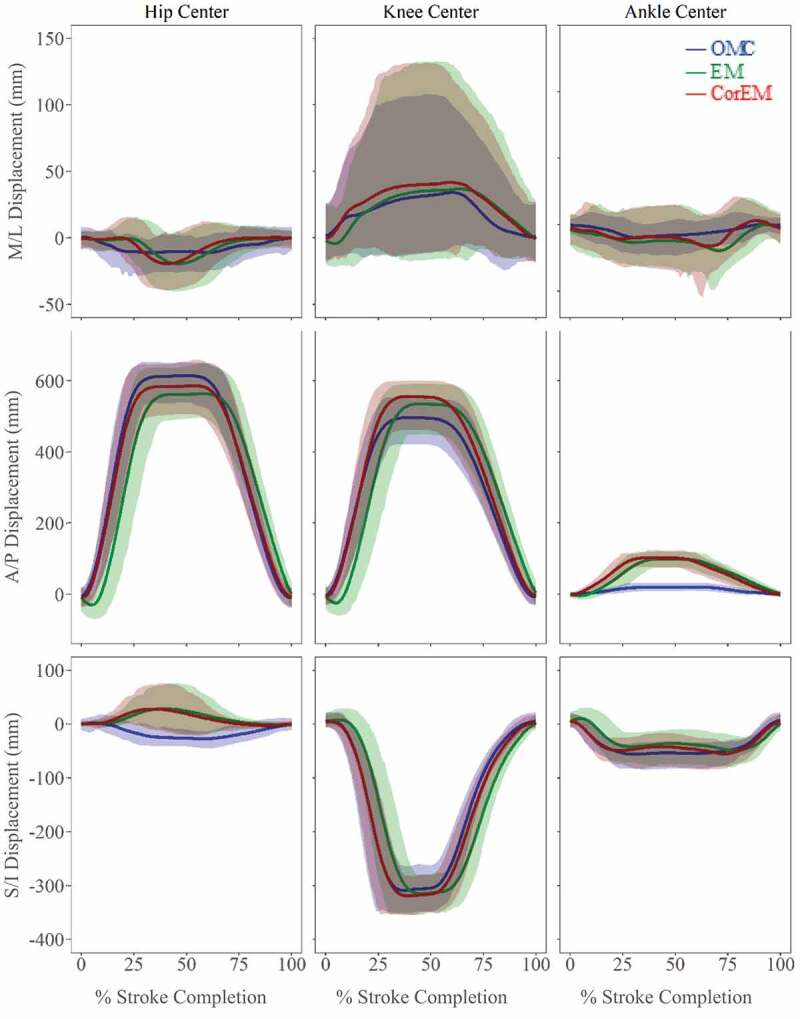
Simultaneous joint center tracking using the three methods (mean ± 95% CI). A/P and S/I displacements show improved trajectory agreement with phase-lag correction. S/I hip joint movement and A/P ankle joint movement show confidence intervals, which do not substantially overlap, with both having large magnitude discrepancies. Confidence of agreement in M/L displacements is ambiguous due to larger relative standard deviations, particularly at knee joint center

GM values (Table 1) across co-localized marker/sensor displacements (Figure 5) and predicted joint centre trajectories (Figure 6) were highly related in A/P direction; however, agreement overall was poorest in M/L direction, with large differences attributed to magnitude. Deviations attributable to phase differences can be substantially reduced by applying phase-lag correction to the EM data, and metric values were good for most trajectories (Table 1 – Phase; *GM: moderate-to-good*), expect for S/I hip joint center, for which GM attributes all deviation to phase effects, despite lag correction. Deviations attributable to magnitude differences show some improvement with phase-lag correction, with good agreement overall in A/P and S/I directions (Table 1 – Magnitude; *GM: moderate-to-good*). However, in the M/L direction, hip and ankle magnitudes improve but were still poor overall, while M/L femur and knee worsen after correction (*GM: moderate-to-poor*). Some of these deviations may be confounded by large confidence intervals present in M/L knee joint center (Figure 6), femur receiver, and tibia receiver (Figure 5).

**Table 1.** Geers metric for agreement between tracking systems as a function of magnitude and phase differences. Values of <0.3 are good (green), between 0.3 and 0.5 moderate (yellow), and >0.5 has poor agreement (red). Values for directly tracked markers/receivers and calculated joint centers, before and after phase-lag correction, are shown. Deviations attributed to phase were reduced by applying phase-lag correction. Negative metric values indicate an under-prediction of EM displacements relative to OMC. Analytical formulations of Geers metric may be found in Schwer (2007)

	Medial/Lateral		Anterior/Posterior		Superior/Inferior	
	Magnitude					
	Original	Phase Corrected	Original	Phase Corrected	Original	Phase Corrected
Lumbar	−0.367	−0.368	−0.040	0.003	−0.300	−0.277
Pelvis	0.266	0.266	−0.027	0.020	−0.307	−0.279
Femur	1.213	1.392	−0.045	0.007	−0.126	−0.095
Tibia	0.221	0.268	0.054	0.100	0.469	0.499
Hip	0.528	0.503	−0.056	−0.009	−0.016	−0.043
Knee	0.253	0.335	0.060	0.108	0.033	0.048
Ankle	1.482	0.998	4.335	4.557	−0.193	−0.065
	**Phase**					
Lumbar	0.456	0.459	0.070	0.016	0.114	0.034
Pelvis	0.172	0.147	0.071	0.016	0.107	0.039
Femur	0.215	0.172	0.073	0.018	0.090	0.024
Tibia	0.261	0.234	0.068	0.015	0.107	0.065
Hip	0.324	0.315	0.072	0.016	0.748	0.687
Knee	0.172	0.115	0.068	0.016	0.083	0.021
Ankle	0.237	0.227	0.066	0.057	0.123	0.062

Rowing is predominately defined by motion in the A/P direction, with large displacements and smaller relative variance yielding a high signal-to-noise ratio. Displacement ranges in M/L and S/I directions were smaller with larger relative standard deviations (Figures 5, 6). Caution is warranted when analyzing small displacements and angular rotations. OMC is more sensitive to small perturbations in marker position, and lower resolution makes EM less sensitive to true shifts.

## Discussion

This study assessed the ability of OMC and EM systems to represent ground truth position in static and dynamic apparatus tracking and the precision and accuracy of those systems when tracking volunteers performing whole-body ergometer rowing. While previous work has compared simple, well-defined movements, tracking systems are increasingly utilized in highly sensitive and complicated performance analyses (Koivukangas et al. 2013; Pueo and Jimenez-Olmedo 2017) where accuracy and precision are paramount.

Accuracy in OMC systems is dependent on appropriate positioning of cameras to observe marker movement and on the quality of calibration of the capture volume. Accuracy in EM systems is dependent on the distance between transmitter and receivers, number of receivers in use, and ferromagnetic interference in the capture volume. Similar studies comparing the accuracy of EM and OMC systems have been conducted utilizing markers/receivers applied to a robotic, articulated arm where the true range of motion was known (Hassan et al. 2007; Lugade et al. 2015). Hassan et al. (2007) concluded that accuracy of EM and OMC systems were comparable for measuring simulated upper extremity kinematics when appropriate post-hoc filtering and corrections were applied.

**Figure 7.**
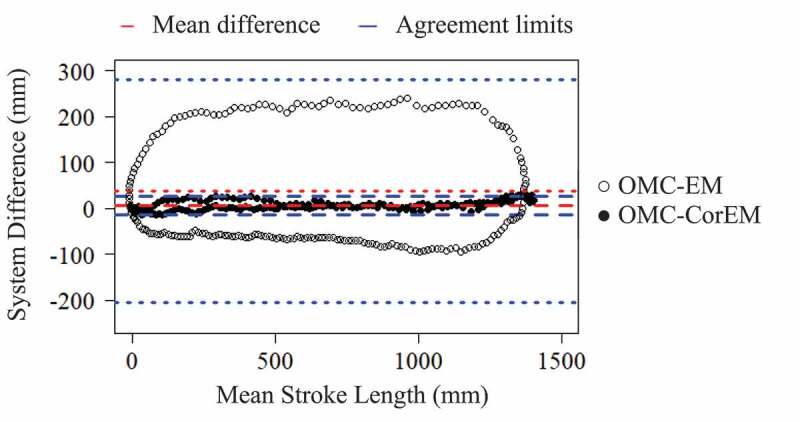
Bland-Altman comparison of bias and limits of agreement (mean ± 95% CI) during dynamic apparatus tracking for A/P handle displacements between OMC and EM systems before (OMC-EM) and after (OMC-CorEM) phase-lag correction. After phase-lag correction the cyclic trend collapses and the limits of agreement contract

The average bias, or discrepancy between tracking methods, was sufficient to be important for assessing motion in multiple planes. If the limits of agreement had been narrow and the bias small, the two methods could be considered essentially equivalent; however, this was not the case between the OMC and EM systems tested here. Examining A/P handle position by Bland-Altman analysis (Figure 7), before phase-lag correction (OMC-EM), there was a clear cyclic trend, where the absolute value of bias increases as mean stroke length increases (mean bias (limits) = 28.1 (−220.3, 276.6) mm). After correcting for the EM system capture latency (OMC-CorEM), this cyclic trend disappears, the limits of agreement contract (mean bias (limits) = 2.2 (−27.3, 31.7) mm) and the variance around the bias line become consistently flat (Figure 7).

After phase-lag correction of the EM system data, the sensor positions reported by both systems tended to fall within 95% confidence interval of each other, suggesting that either system is sufficiently accurate when the signal-to-noise ratio is high, as was the case for large A/P movements (Figures 5, 6). Both systems tended to have difficulty accurately tracking smaller M/L movements (Figure 6). A similar magnitude effect was reported by Hassan et al. (2007) where deviations from the ‘ground truth’ articulated robotic arm position occurred mostly at small angles and displacements. The authors did not assess repeatability of the tracking systems using an *in vivo* model. Lugade et al. (2015) reported sagittal plane lower limb joint angles during a sit-to-stand activity, finding that OMC had lower RMS errors and higher intra- and inter-day CMC values (0.99 ± 0.001; 0.97 ± .025) than EM (0.97 ± 0.05; 0.94 ± 0.038) but concluded that both systems were adequate for tracking dynamic motion (Lugade et al. 2015).

Assessment of accuracy and precision of EM systems in the context of rowing kinematics have been reported (Bull and McGregor 2000; Ng et al. 2009). Ng et al. (2009) limited their study to the assessment of a static multi-hinge model being moved with constant rate (22 spm) along an ergometer slide rail but suggested that different velocities may elicit variations in error. While sequential data transmission of EM receivers could be detrimental to data acquisition of higher rate movements, this does not appear to have affected the current study. Increasing from 18 spm to 28 spm did not translate into changes in mean position differences, nor in the data variance. Ng et al. (2009) also noted that of the four receivers employed during their experiments, the receiver located farthest from the transmitter produced the largest angular error, while the receiver closest to the transmitter produced the smallest angular error. This is consistent with results from the current study’s variability in ergometer seat width as receivers were moved along the slide rail past the transmitter, with estimates deviating more from the true value as the radial distance of each receiver to the transmitter increased (Figure 4).

Bull and McGregor (2000) demonstrated that an EM device was sufficiently accurate to discern differences in rowing technique and movement patterns in the sagittal plane, with reported spinal parameters used to discriminate between qualitatively ‘good’ and ‘bad’ rowing technique. Results from the current study appear to support the conclusion that the EM system is capable of discerning salient changes in A/P trajectories (Figure 5), due to displacements in the rowing stroke being very large in the A/P direction compared to the resolution of the receivers. M/L and S/I displacements during rowing were relatively small, and many of the Geers metric magnitude values were moderate-to-poor in the M/L direction. Given reported difficulties in analyzing small displacements and angular rotations using an EM system (Hassan et al. 2007), discriminating differences in spinal torsion and lateral bending may not be feasible, and re-examining published spinal flexion/extension values may be future work.

### Limitations

The left seat EM receiver was thus farther from the EM transmitter than the right seat EM receiver. Despite a published functional radius of up to 1.5 m for the EM system, tracking of movement near the *catch* and the *release* positions may have been affected by the greater radial distance from the transmitter, contributing to the discrepancy in A/P seat position between left and right receivers and in greater error estimating seat width. Ng et al. (2009) noted that differences in ferrous material content in different parts of the ergometer may contribute to variability of the data.

Anatomical variation among participants, biomechanical differences in performing the rowing task, and skin motion artifact could also have affected results for both systems. Digitization differences at the start of testing were minimized by using one well-trained operator for marker placement, who was an experienced sports biomechanist and physiotherapist with substantial training in landmark palpation, skin marker placement and specific experience with methods used in the current protocol. However, OMC markers and EM receivers can shift during testing, introducing error in anatomical landmark position. Intrinsic kinematics of rowing regularly cause occlusion of OMC markers fixed to the anterior pelvis, and relying on digital reconstruction may be a source of error.

## Conclusions

This study specifically compared the dynamic accuracy and precision of EM and OMC displacements in three dimensions during ergometer rowing by simultaneous motion tracking. While both EM and OMC systems demonstrated an ability to adequately track large dynamic compound movements in the sagittal plane during ergometer rowing, both systems struggled to precisely track small displacements in M/L and S/I directions. OMC outperformed EM tracking for salient measurements of precision, speed, and consistency in data acquisition. Expanded capture capabilities with OMC are beneficial for whole-body motion studies and justify further use of OMC without losing the ability to compare to prior work, as system compatibility facilitates interpretation of future OMC data in relation to EM measures.
